# Predicting the Skin Sensitization Potential of Small Molecules with Machine Learning Models Trained on Biologically Meaningful Descriptors

**DOI:** 10.3390/ph14080790

**Published:** 2021-08-11

**Authors:** Anke Wilm, Marina Garcia de Lomana, Conrad Stork, Neann Mathai, Steffen Hirte, Ulf Norinder, Jochen Kühnl, Johannes Kirchmair

**Affiliations:** 1 Center for Bioinformatics (ZBH), Department of Informatics, Universität Hamburg, 20146 Hamburg, Germany; wilm@zbh.uni-hamburg.de (A.W.); stork@zbh.uni-hamburg.de (C.S.); 2 HITeC e.V., 22527 Hamburg, Germany; 3 Department of Pharmaceutical Sciences, Faculty of Life Sciences, University of Vienna, 1090 Vienna, Austria; a11853333@unet.univie.ac.at (M.G.d.L.); steffen.hirte@univie.ac.at (S.H.); 4 Computational Biology Unit (CBU), Department of Chemistry, University of Bergen, N-5020 Bergen, Norway; neann.mathai@uib.no; 5 MTM Research Centre, School of Science and Technology, Örebro University, SE-70182 Örebro, Sweden; ulf.norinder@farmbio.uu.se; 6 Department of Computer and Systems Sciences, Stockholm University, SE-16407 Kista, Sweden; 7 Department of Pharmaceutical Biosciences, Uppsala University, SE-75124 Uppsala, Sweden; 8 Front End Innovation, Beiersdorf AG, 22529 Hamburg, Germany; Jochen.Kuehnl@Beiersdorf.com

**Keywords:** skin sensitization, toxicity prediction, in silico prediction, machine learning, random forest, conformal prediction, bioactivity descriptors

## Abstract

In recent years, a number of machine learning models for the prediction of the skin sensitization potential of small organic molecules have been reported and become available. These models generally perform well within their applicability domains but, as a result of the use of molecular fingerprints and other non-intuitive descriptors, the interpretability of the existing models is limited. The aim of this work is to develop a strategy to replace the non-intuitive features by predicted outcomes of bioassays. We show that such replacement is indeed possible and that as few as ten interpretable, predicted bioactivities are sufficient to reach competitive performance. On a holdout data set of 257 compounds, the best model (“Skin Doctor CP:Bio”) obtained an efficiency of 0.82 and an MCC of 0.52 (at the significance level of 0.20). Skin Doctor CP:Bio is available free of charge for academic research. The modeling strategies explored in this work are easily transferable and could be adopted for the development of more interpretable machine learning models for the prediction of the bioactivity and toxicity of small organic compounds.

## 1. Introduction

Substances that can induce allergic contact dermatitis after repeated contact to the skin are called skin sensitizers [1,2]. In order to prevent the induction of skin sensitization, exposure to skin sensitizers must be minimized [3,4,5,6,7,8]. The ability to detect and predict skin sensitizers is therefore of significant importance for several sectors of industry to develop safe and efficacious functional small molecules [9].

Until recent years, strategies to assess the risk of small molecules to induce skin sensitization relied on animal experiments. Historically, an important animal experiment to address skin sensitization potential is the guinea pig maximization test (GPMT), which was used to determine the percentage of test animals that develop contact allergy symptoms after repeated exposure to the test substance. Typically, a substance was classified as a sensitizer if at least 15% of the guinea pigs developed allergic symptoms. The GPMT was later replaced by the murine local lymph node assay (LLNA) [10], an animal model measuring the proliferation rate of cells in the draining lymph node in mice. The LLNA is still regarded as the gold standard among the animal experiments to assess skin sensitization potential as it provides advantages concerning animal welfare (compared to other animal models) and additional information to quantify the skin sensitization potency of compounds (based on the EC3 value, defined as the substance concentration that induces a 3-fold stimulation of proliferation) [11,12].

Ambitious efforts are ongoing to fully replace animal experiments, and a diverse set of alternative experimental and theoretical methods have been developed [13,14] to assess skin sensitization potential and, to a limited degree, skin sensitization potency [15]. Among others, these approaches include non-animal testing methods (i.e., in vitro and in chemico assays) [16,17,18,19] and in silico methods [18,19,20,21].

Several OECD-validated non-animal testing methods address three out of four key events of the adverse outcome pathway of skin sensitization induction: The first key event, or molecular initiating event, describes the so-called haptenization, which is the covalent binding of the substance to skin proteins or peptides. This is experimentally assessed by the direct peptide reactivity assay (DPRA) [22]. The second key event, which is the activation of keratinocytes [23], is covered by the KeratinoSens and LuSens assays, while the third key event, which is the activation of the skin’s dendritic cells [24], is addressed, among others, by the U937 cell line activation test (U-SENS) and the human cell line activation test (h-CLAT). As all of these assays cover certain aspects of the adverse outcome pathway; none of them is suitable as a standalone methodology for the prediction of the skin sensitization potential of small molecules.

Computational methods that predict skin sensitization can be classified into expert systems, similarity-based approaches, and (quantitative) structure–activity relationship (QSAR) approaches [20]. These approaches offer fast predictions at low cost, enabling their use also in early stages of research and development, where a large number of candidate compounds may be under investigation. To be accepted as a component of regulatory risk assessment, computational methods have to fulfill certain quality criteria. For example, according to the OECD [25], a model should have a defined endpoint, an unambiguous algorithm, a defined applicability domain, appropriate measures of goodness-of–fit, robustness, and predictivity, and, if possible, a mechanistic interpretation.

No particular non-animal testing method or individual computational model has so far yielded a level of performance, robustness, interpretability, and coverage to be accepted as a standalone approach for skin sensitization prediction in the regulatory context. The most promising strategy to advance alternative testing methods is the combination of experimental and computational tools [26] within defined approaches, integrated approaches for testing and assessment (IATAs; for a review of IATAs and defined approaches see ref. [27]), or in “weight of evidence” considerations [28].

In our previous work [29], we presented Skin Doctor CP, a random forest (RF) model for the prediction of LLNA outcomes for small molecules that complies with the above-mentioned OECD principles to the furthest possible extent. The Skin Doctor CP model is trained on a set of 1278 compounds annotated with binary LLNA outcomes (i.e., skin sensitizer and skin non-sensitizer). To the best knowledge of the authors, this data set represents the largest collection of high-quality LLNA data in the public domain at present. The data set has been characterized regarding its composition and chemical space coverage [29]. The RF model derived from this data set is wrapped into an aggregated Mondrian conformal prediction (CP) framework, which ensures predictivity and robustness by a mathematically founded measure of reliability [30,31,32]. More specifically, the CP framework guarantees an observed prediction error of the model close to the error rate ε set by the user (this is as long as the randomness assumption of the samples holds true; an assumption that is also made for any classical machine learning model). The CP framework will only return a predicted class membership for a substance if the prediction lies within the desired confidence level 1-ε. The measure of reliability offered by the CP approach can guide the use of Safety Assessment Factors of different levels and serve as a powerful, mathematically founded alternative to applicability domain definitions [33].

Depending on the available data and computational capacities, different variants of CP may be developed [34]. In the case of LLNA prediction, the data available for model development are limited and imbalanced; hence, the use of an aggregated CP framework is advised. The aggregated CP framework repeats the framework several times with different proper training and calibration sets [35]. This reduces the variance in the model predictions and allows every datapoint of the training set to be used for model development. It is therefore best suited for modeling small data sets.

To address data imbalance in addition to data scarcity (such as in the case of the LLNA data modeled in our previous study), the combination of the aggregated CP framework with Mondrian CP is advised. Mondrian CP is tailored to describe imbalanced data as it treats each of the classes independently and ensures the validity of their predictions [36,37,38]. This is especially beneficial in toxicity prediction, where the toxic class is usually the minority class and therefore more difficult to predict [39].

In addition to the OECD requirement for a model to produce results with defined reliability (which we address by using a CP framework), model interpretability is a further key factor to consider. Model interpretability depends on the types of descriptors employed in model building. Most of the existing models for the prediction of the skin sensitization potential of compounds, including our Skin Doctor CP models, rely on molecular fingerprints [29,40,41,42]. Interpreting these fingerprints can prove challenging, but in general, some links between chemical patterns and the biological outcomes can be identified [43].

In an attempt to generate predictive models from physically meaningful (and hence more intuitive) descriptors, we previously investigated the capacity of physicochemical property descriptors to produce predictive models for the prediction of the skin sensitization potential [44]. However, the models trained on physicochemical property descriptors do not perform as well as those trained on molecular fingerprints, and their interpretation is still challenging due to the high number of descriptors required to obtain models with an acceptable performance.

Recent studies have shown that in silico models for the prediction of complex in vivo endpoints can benefit from the inclusion of measured or predicted biological data (i.e., in vivo and/or in vitro data) into the feature set. More specifically, descriptive models have been built on small sets of hand-picked biological descriptors relevant to the endpoint of interest [45], as well as on large sets of screening data that may or may not be directly related to the endpoint of interest [46,47,48,49,50]. There are several examples of in silico models, nearest neighbor approaches in particular, that are trained on predicted bioactivities [51,52]. For example, the RASAR models [53] are RF models that predict nine health hazard endpoints (including the skin sensitization potential) based on the distances of a compound of interest to its nearest active and inactive neighbors in reference data sets for 19 toxicological outcomes. Another computational approach utilizes a reasoning framework to build an information-rich network based on assay knowledge, assay data, and predicted bioactivities [54]. The visualization of this network can provide guidance to researchers for the assessment of the safety profile of small molecules.

Recently, Norinder et al. [55] presented a CP framework that utilizes predicted bioactivities as input for in silico models for bioactivity and cytotoxicity prediction. This approach has the advantage of improving a model’s predictivity by the use of bioactivity data without the need to perform additional experimental testing for a compound of interest. A similar methodological framework was successfully applied to three in vivo toxicological endpoints (i.e., genotoxicity, drug-induced liver injury, and cardiological complications) by some of us [56].

The aim of this work is to investigate the capacity of predicted bioactivities to produce simple, interpretable machine learning models for the prediction of the skin sensitization potential of small organic compounds without compromising on performance. In order to reach this goal, we explored strategies to replace the molecular fingerprints (MACCS keys) used in Skin Doctor CP by a small set of predicted bioactivities. We selected these predicted bioactivities using Lasso regression from a panel of 372 published CP models for compound toxicity prediction [56] plus three new, additional models for assays of direct relevance to skin sensitization (i.e., DPRA, KeratinoSens assay, and h-CLAT). The final classifiers for the prediction of the skin sensitization potential of compounds were trained on 1021 compounds. They utilize only 10 predicted bioactivity descriptors but perform comparably to the Skin Doctor CP models. The best model (“Skin Doctor CP:Bio”) is available free of charge for academic research purposes.

## 2. Materials and Methods

### 2.1. Data Sets and Data Processing

#### 2.1.1. Binary LLNA Data

This work is based on the identical LLNA data set that was used for the development of Skin Doctor CP [29]. The random split into a training set (80%) and a test set (20%) was also preserved. The chemical structures were processed with a refined preprocessing protocol that was developed by Garcia de Lomana et al. [56]. This protocol includes the removal of solvents and salts, annotation of aromaticity, neutralization of charges, and mesomerization. Substances containing (i) different components with non-identical SMILES or (ii) fewer than four heavy atoms or (iii) elements other than H, B, C, N, O, F, Si, P, S, Cl, Se, Br, and I were removed from the data set.

The use of the new structure preprocessing protocol led to the rejection of 7 compounds of the training set (and none of the test set) because they do not fulfill the requirements for molecules to be composed of at least one carbon atom and to consist of at least four heavy atoms. The processed training set consists of 1021 compounds and the test set of 257 compounds.

#### 2.1.2. Non-Animal Data on Skin Sensitization

For the calculation of additional bioactivity descriptors, chemical information, and binary assay data for 194 compounds measured in the DPRA, 190 compounds measured in the KeratinoSens assay and 160 compounds measured in the h-CLAT were collected from Alves et al. [57]. The chemical structures were preprocessed following the protocol described above. Preprocessing resulted in the removal of one particular substance (formaldehyde) that is present in all three data sets. The final KeratinoSens assay, h-CLAT, and DPRA data sets comprised 189, 159, and 193 compounds, respectively.

#### 2.1.3. Data for Chemical Space Analysis

In preparation for chemical space comparison, the 7030 cosmetics and 4036 agrochemicals included in the CompTox Chemicals Dashboard [58] and the 2509 approved drugs included in DrugBank [59] were downloaded and processed following the protocol described above. This resulted in a data set of 4488 cosmetics, 2433 agrochemicals, and 2227 approved drugs (the significant reductions are related to the fact that many of the listed cosmetics and agrochemicals are either inorganic salts or without a defined molecular structure).

### 2.2. Descriptor Calculation and Normalisation

A set of 750 bioactivity descriptors related to 375 predicted binary assay outcomes was calculated for all compounds of the LLNA data set and the three reference data sets (the number of bioactivity descriptors is double that of the predicted binary assay outcomes because the predicted class probabilities of the active and the inactive class were included in the descriptor set independently from each other). More specifically, class probabilities for 372 bioactivity assays were calculated with aggregated Mondrian CP models that we trained on bioactivity assay data collected from ToxCast [60], eMolTox [61], the eChemPortal [62], and literature, following the identical protocol published by Garcia de Lomana et al. [56]. In addition, predicted class probabilities for three assays relevant to skin sensitization prediction (i.e., DPRA, KeratinoSens assay, h-CLAT) were computed using Mondrian CP models generated by applying the identical model generation framework as described for the other assays [56] to the three corresponding data sets retrieved from Alves et al. Prior to modeling, the standard scaler of the preprocessing module of scikit-learn [63] was used (with default settings) to normalize all bioactivity descriptors. The standard scaler was trained on the LLNA training set only and applied to the full LLNA data set (training and test set). In addition, MACCS keys were calculated with RDKit version 2020.09.1 [64] for all compounds in the LLNA data set.

### 2.3. Model Development

#### 2.3.1. Aggregated Mondrian Conformal Prediction Modeling

In preparation for model generation, each training set was divided into a proper training set (80%) and a calibration set (20%) by stratified random splitting utilizing the train_test_split function of the Model_selection module of scikit-learn (data shuffling was enabled prior to data set splitting). Then, a RF model was generated (with the RandomForestClassifier function of scikit-learn; all parameters kept default, except for n_estimators = 500 and random_state = 43) and applied to the corresponding calibration and test set.

From the prediction probabilities obtained for the calibration set and the test set, non-conformity scores (*α*-values) were calculated following Equation (1):

(1) $$\alpha_{i}=0.5-\frac{\hat{P}\left(y_{i}|x_{i}\right)-max_{y\neq y_{i}}\hat{P}(y|x_{i})}{2}$$

where $\hat{P}\left(y_{i}|x_{i}\right)$ is the class probability for class *i* returned by the model, and $max_{y\neq y_{i}}\hat{P}\left(y|x_{i}\right)$ is the maximum class probability for any other class returned by the model.

The non-conformity scores of the calibration set were sorted class-wise (following the Mondrian conformal prediction protocol), and the relative ranks of the non-conformity scores of each compound of the test set in relation to these lists were retrieved as so-called *p*-values.

Within the aggregated CP framework, the procedure was repeated for 20 times with different stratified random splits into a proper training and calibration set, altering the random state of the train_test_split function from 0 to 19. For every compound in the test set, a *p*-value was derived during each run. The median over the *p*-values obtained during all 20 runs was processed as the final *p*-value of the compound. The *p*-values denote the probability of a compound belonging to the corresponding activity class. The model assigns a compound to a specific activity class if the corresponding *p*-value exceeds the selected error significance level ε.

#### 2.3.2. Measurement of Model Performance

In this work, the classical performance measures (i.e., accuracy (ACC), Matthews correlation coefficient (MCC) [65], correct classification rate (CCR), sensitivity (Sens), specificity (Spec), negative predictive rate (NPV), and positive predictive rate (PPV)) are calculated based exclusively on compounds that were assigned by the CP models to exactly one activity class, i.e., “sensitizer” or “non-sensitizer”. This is to enable the application of classic performance measures to CP and, at the same time, to ensure the comparability of the classical performance measures and the results reported for classical non-CP models elsewhere.

In contrast, the CP-specific performance measures (i.e., validity and efficiency) are calculated for all models based on the full sets of compounds to fulfill the common definition of these measures and enable the comparison with other CP models. Validity is defined as the percentage of predictions that include the true class, independently of the prediction of the other class (i.e., it includes “true” predictions as well as “both” predictions). A model is deemed to be valid if the validity is close or equal to the expected value of 1-ε. Efficiency can be understood as an equivalent to the term coverage for non-CP models. It is defined as the percentage of distinct predictions (i.e., predictions that predict exactly one class to be true).

#### 2.3.3. Feature Selection and Parameter Optimization

**Figure 1:**
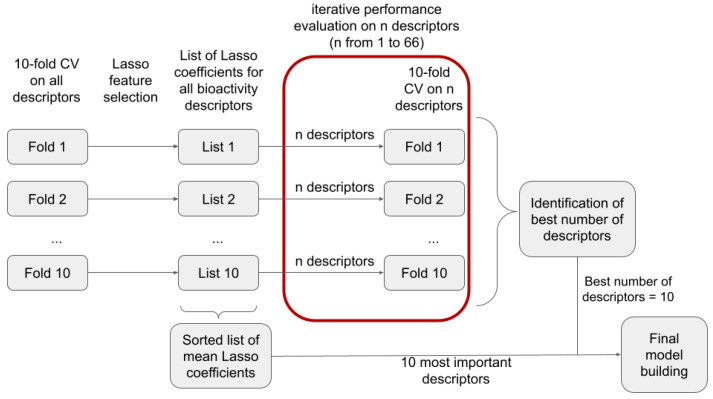
Schematic representation of the workflow for feature selection.

For feature selection (Figure 1), 10-fold cross-validation (CV) was performed on the training set using the scikit-learn StratifiedKFold function (Model_selection module; n_splits = 10, shuffle = True, random_state = 43).

First, the relative importance of each feature within each fold of the CV was investigated. Therefore, hyperparameters for a Lasso classifier were optimized by a 10-fold CV within each fold of the outer CV. This was achieved with the scikit-learn LassoCV function (Linear_model module; random_state = 43, cv = 10, max_iter = 3000, n_alphas = 200). The optimized Lasso classifier was then used to obtain the Lasso coefficients of all bioactivity descriptors within the corresponding fold. The relative importance of each descriptor was calculated as the absolute value of the mean Lasso coefficient calculated over all folds of the CV run.

Second, the optimum number of bioactivity descriptors for model generation was determined. To do so, the 10-fold CV on the training data was repeated, this time without feature selection with Lasso. Instead, a varying number of the most important bioactivity descriptors (i.e., 1 to 66 descriptors; selected based on their coefficients obtained with Lasso) were selected for model building. The mean performance during 10-fold CV in dependence of the number of descriptors was used to select the number of features for the final model.

## 3. Results and Discussion

### 3.1. Identification of the Optimum Number of Bioactivity Descriptors for Model Building

**Figure 2:**
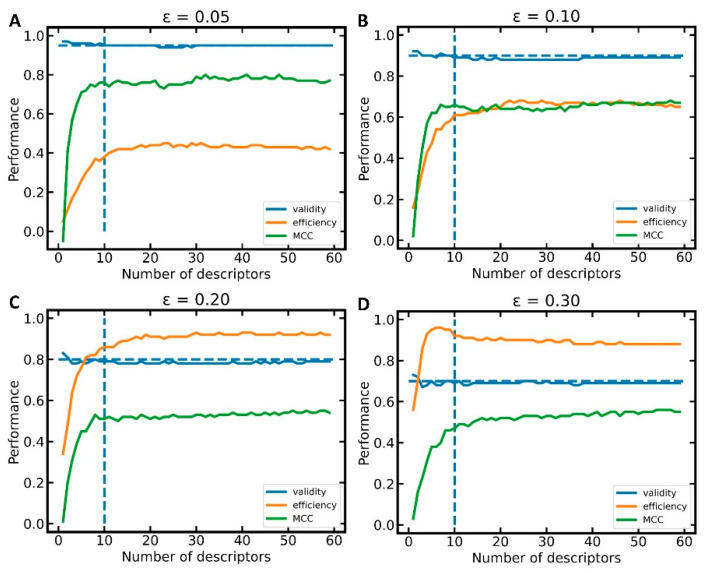
Mean performance of 10-fold CV as a function of the number of bioactivity descriptors selected for model building at the significance level (**A**) ε=0.05, (**B**) ε=0.10, (**C**) ε=0.20, (**D**) ε=0.30. The horizontal, dashed line indicates the validity expected from the selected significance level ε; the vertical, dashed line marks the performance of models trained on 10 descriptors.

In order to identify the most suitable number of bioactivity descriptors *n* for model building, we investigated, within a 10-fold CV framework, the performance of models as a function of the number of descriptors used (reflecting model interpretability/complexity). Within each CV fold, we performed Lasso regression to rank the descriptors by their corresponding Lasso coefficients (Table S1) and selected the *n* most important descriptors for model building. In Figure 2, we show the improvement of model performance as more bioactivity descriptors are added. In particular, for the first 10 descriptors, a steep increase in MCC and efficiency is observed (see section “Measurement of model performance” of the Methods for important information on how, and in particular on what data, the individual performance measures are calculated). Beyond 10 descriptors, the improvements in model performance are minor and reach a plateau at approximately 25 descriptors. This led us to the conclusion that models based on the 10 most relevant bioactivity descriptors offer the best balance between model performance and complexity (Table 1). Validity is close to the expected value of 1-ε for all the significance levels (i.e., ε = 0.05, 0.10, 0.20, and 0.30) and numbers of descriptors (in this experiment, 1 to 66) investigated.

### 3.2. Investigation of the Ten Most Relevant Bioactivity Descriptors

With 10 identified as the optimum number of bioactivity descriptors for model building, we reiterated the above-mentioned descriptor selection process on the full training set and analyzed the relevance and biological meaning of the 10 descriptors with the highest absolute Lasso coefficients averaged over the 10 folds of the CV (Table 2).

The bioactivity descriptor ranked first by the Lasso model is the ToxCast assay “BSK KF3CT ICAM1 down” (Lasso coefficient 0.074). This feature describes the expression of ICAM1 in human keratinocytes. This ToxCast assay is observed to correlate with predictions for other keratinocytes and foreskin assays from the ToxCast BSK family (Kendall τ correlation coefficients between 0.77 and 0.79). The ICAM1 readout is also known as CD54, which is a readout of the skin sensitization-related h-CLAT. The underlying model shows good predictivity (validity = 0.80, efficiency = 0.83, MCC = 0.41 at the significance level of 0.20) The nine further bioactivity descriptors all have similar Lasso coefficients, between 0.036 and 0.051 (validities between 0.74 and 0.87; efficiencies between 0.51 and 0.87; MCCs between 0.30 and 0.98, respectively). Among these are the three assays that we added to the descriptor set because of their direct relevance to skin sensitization: DPRA, KeratinoSens assay, and h-CLAT. As expected, a direct correlation between a positive outcome in any of these three assays and the probability of a compound being a skin sensitizer is identified by the Lasso model. The fact that these assays do not show a high correlation with any other bioactivity descriptors within our full set of descriptors underlines the fact that these descriptors may add important additional information on the skin sensitization potential of compounds. The models predicting these bioactivity descriptors are built on comparably small data sets (<200 compounds). This is reflected by a higher deviation of the significance of these models from the expected value of 0.80 at the investigated significance level of 0.20, compared to the other models. The MCCs of these models are between 0.30 and 0.54.

The ToxCast assay “ATG NRF2 ARE CIS up” describes the activation of NRF2 in human liver cells. Being the fundamental concept of keratinocyte activation analysis via KeratinoSens and LuSens assay, Nrf2 activation is known to play a vital role in the regulation of cellular cytoprotective responses, metabolism, and immune regulation. Included in the top-10 features are also the ToxCast assays “BSK 3C E-selectin down” and “BSK 4H uPAR down”, both of which describe inflammation-related biological processes in the endothelium environment. As such, these assays might encode aspects of the immunological response of the human body. “BSK 3C E-selectin down” correlates with other assays associated with inflammation and immune reaction and which are often located in the endothelium. While it shows a positive correlation with the skin sensitization potential (which might indicate an activation of compounds or increased bioavailability), “BSK 4H uPAR down” is one out of only two bioactivity descriptors (among the top-10 features) that show negative correlation with the skin sensitization potential. This assay may therefore report processes involving the deactivation of a compound or the reduction of its bioavailability.

The chromosome aberration assay may not be directly linked to skin sensitization, but it may be relevant to the detection of reactive compounds. The feature is weakly correlated with other assays that are linked to the detection of reactive molecules (e.g., mammalian cell gene mutation assay or AMES mutagenicity assay). Chromosome aberration predictions show no strong correlation with any other descriptors in the set of models.

### 3.3. Coverage of the Chemical Space Relevant to the Development of Cosmetics, Drugs and Agrochemicals

**Figure 3:**
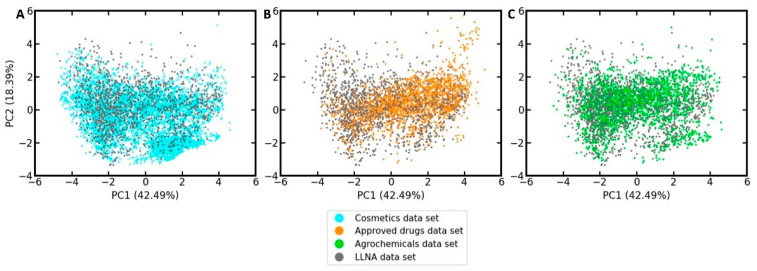
PCA quantifying the coverage of the LLNA data by the reference sets of (**A**) cosmetics, (**B**) approved drugs, and (**C**) agrochemicals in the feature space of the 10 selected bioactivity descriptors. The percentages in parentheses report the variance explained by the respective principal component (PC).

In order to develop an understanding of to what extent the LLNA data set, which we will use to develop the in silico models, represents drugs, cosmetics, and agrochemicals in the feature space defined by the ten selected bioactivity descriptors, a principal component analysis (PCA) was performed on the LLNA data set and the reference sets. As shown in the PCA scatter plot in Figure 3 (PCA loadings plot provided in Figure S1), the LLNA data set covers well the areas in feature space populated by cosmetics, approved drugs, and agrochemicals.

### 3.4. Analysis of the Distribution of Sensitizers and Non-Sensitizers in the Feature Space of the Ten Selected Bioactivity Descriptors

**Figure 4:**
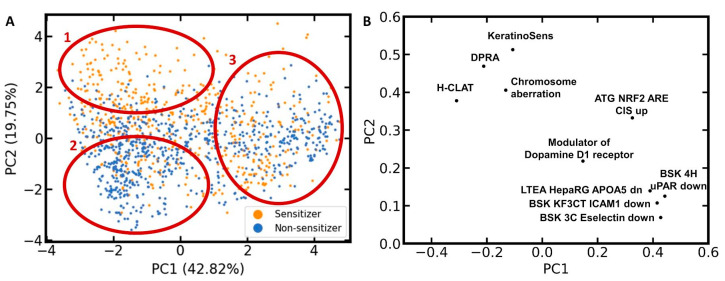
LLNA data set analyzed by PCA in the feature space of the ten selected bioactivity descriptors. (**A**) Scatter plot colored by the binary skin sensitization potential; (**B**) loadings plot of the ten descriptors. The percentages in parentheses report the variance explained by the respective principal component (PC). Note that the axis sections differ for panels (**A**,**B**).

To investigate the distribution of sensitizers and non-sensitizers within the feature space of the ten selected bioactivity descriptors, another PCA was performed, this time exclusively on the compounds of the LLNA data set (Figure 4). Three characteristic areas can be identified in the scatter plot resulting from this PCA (Figure 4A): Area 1, covering mainly sensitizers; Area 2, covering mainly non-sensitizers; and Area 3, showing intense mixing of sensitizers and non-sensitizers.

The corresponding loadings plot (Figure 4B) places the bioactivity descriptors for the three skin sensitization assays (h-CLAT, DPRA, and KeratinoSens assay) and the chromosome aberration assay in quadrant 2 (upper left). All four of these assays contribute positively to PC2 and, to a lower degree, negatively to PC1. Since a positive outcome in one or several of the skin sensitization assays should be correlated with a positive skin sensitization potential, this is in agreement with the PCA scatter plot showing a high accumulation of sensitizers in the upper left region. Since a positive outcome in the chromosome aberration assay is likely correlated with a reactive compound, it is also within the expectations that it will shift a compound towards this Area 1 in the PCA scatter plot.

For the remaining six bioactivity descriptors, higher PC1 and PC2 values are expected for compounds that are active in the corresponding assay. Thus, all ten bioactivity descriptors contribute positively to PC2. This means that every compound predicted to be positive in those bioactivity assays is moved towards Area 1 or 3 in the scatter plot. This comes along with the increased probability of a compound to be a skin sensitizer (i.e., to be located in Area 1). At the same time, every negative predicted assay outcome moves the compound towards Area 2, where we mainly expect non-sensitizers to be located, or Area 3, where no prevalence in activity is detected. This positive contribution to PC2 is higher for KeratinoSens, DPRA, chromosome aberration, h-CLAT, and ATG NRF2 than for the other five bioactivity descriptors. In Area 3, we observe intense mixing of skin sensitizers and non-sensitizers, hence posing a significant challenge to classification.

### 3.5. Model Based on Ten Selected Bioactivity Descriptors

Following the identification of the optimum model setup, a final, aggregated Mondrian CP model based on the ten selected bioactivity descriptors was derived from the full training set and evaluated on the holdout data set. From here on, we refer to this model as the SkinDoctor CP:Bio model.

#### 3.5.1. Performance on the Test Set

Within the standard deviation expected from CV, the SkinDoctor CP:Bio model was valid at all four significance levels investigated (Table 3). The efficiencies of the model ranged from 0.39 to 0.95 and the MCCs ranged from 0.72 to 0.49, depending on the significance level.

Class-wise performance analysis (Table 4) showed that the SkinDoctor CP:Bio model was valid for sensitizers and non-sensitizers at all significance levels investigated. The largest difference in validity between the two classes (0.08) was observed at the significance level of 0.30. Efficiency was in general similar for both classes (largest difference 0.04).

#### 3.5.2. Comparison of the New Model with the Skin Doctor CP Model

The previously developed Skin Doctor CP model [29] is trained on MACCS keys (166 features), whereas the Skin Doctor CP:Bio model is trained on ten selected bioactivity descriptors. All other differences in the data and protocols used for model building and testing are minor (Table S3), thus enabling a direct, comparative assessment of the two feature types and their impact on model performance and behavior.

On the holdout data set of 257 compounds measured in the LLNA (none of these compounds is part of the training set of either model), both the Skin Doctor CP model and the Skin Doctor CP:Bio model were valid at all significance levels investigated. For the sake of clarity, we focus our discussion here on the commonly applied significance level of 0.20; performance data on all significance levels are provided in Table S4. At the significance level of 0.20, the Skin Doctor CP and Skin Doctor CP:Bio models yielded validities of 0.82 and 0.81, respectively. The efficiencies (0.78 vs. 0.82) and MCCs (0.55 vs. 0.53) obtained for the Skin Doctor CP and Skin Doctor CP:Bio models were also comparable. The differences in performance between the two models are slightly above the standard deviation observed for the 10-fold CV experiments but small enough to consider the performance of the two models similar.

### 3.6. Combination of Bioactivity Descriptors with MACCS Keys in an Attempt to Improve Model Performance

MACCS keys encode structural patterns of molecules and thus information that is very different from that encoded by the bioactivity descriptors. The use of MACCS keys in combination with the ten selected bioactivity descriptors could hence yield better models. However, a RF model derived from the combined set of MACCS keys and the ten selected bioactivity descriptors (n_estimators = 500; all other parameters default) did not yield better performance on the test set.

**Figure 5:**
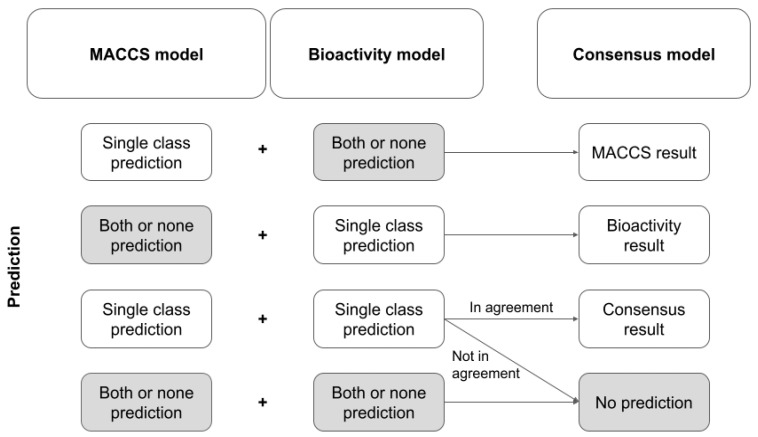
Architecture of the consensus model.

Therefore, we generated a model trained exclusively on MACCS keys plus a model trained exclusively on the ten selected bioactivity descriptors (both models with n_estimators = 500; all other parameters default), and, based on a simple set of rules (see Figure 5), combined both models to form a consensus model. This set of rules follows the idea that only unambiguous predictions by the single models (i.e., predictions assigning a compound to exactly one class) are considered. If one model returns an unambiguous prediction or if both models return an unambiguous prediction and are in agreement, the unambiguous prediction is reported as the final result. In all other cases, the consensus model does not return a prediction.

Table 5 reports on the performance of this consensus model at different error significance levels. Note that because the consensus model does not fulfill the definitions of a pure CP model, validity and efficiency cannot be calculated for this model.

When running the two CP models underlying the consensus approach at a significance level of 0.20, the consensus approach reached a coverage of 0.89 and an MMC of 0.54. Hence, compared to the Skin Doctor CP:Bio model (efficiency 0.82 and MCC 0.53 at a significance level of 0.20), the consensus model obtained only slightly better coverage while maintaining the MCC.

A second, combined, model was constructed by averaging the *p*-values returned for each class by the model based on MACCS keys and the model based on bioactivity descriptors. The model was valid to over-predictive at the four significance levels investigated. At the significance level of 0.20, the validity was 0.82. The efficiency at this significance level was 0.79 (vs. 0.82 for the Skin Doctor CP:Bio model) and the MCC was 0.56 (vs. 0.53 for the Skin Doctor CP:Bio model). Hence, compared to the Skin Doctor CP:Bio model, this combined model obtains a slightly higher MCC, at the cost of efficiency.

**Figure 6:**
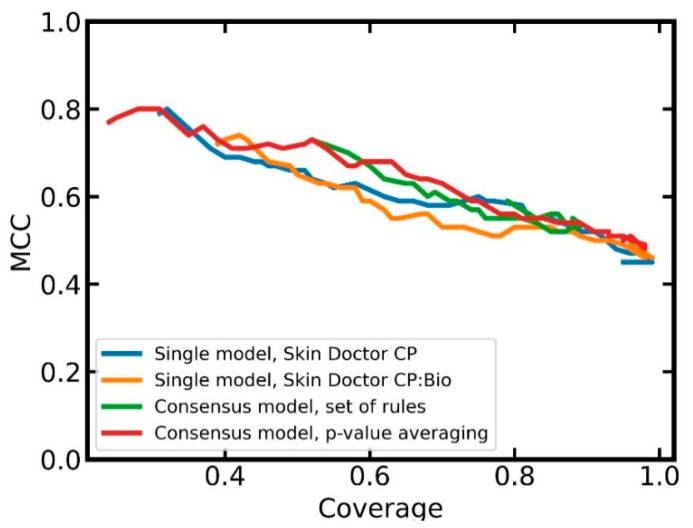
Relationship between MCC and coverage for the individual and the combined models.

In order to obtain a better understanding of the advantages and disadvantages of the two combined models over the single models, we investigated the relationship between classification performance (MCC) and coverage. From Figure 6, it can be seen that the combined models tend to obtain better MCC values at a given coverage than the single models. At higher coverages, the combined model based on averaged *p*-values has slightly better MCCs than the combined model based on the set of rules. A further advantage of the combined model based on *p*-value averaging is that users can select a confidence level; this is not possible with the combined model based on the set of rules.

Overall, the *p*-value averaging approach seems to be preferable over the rule-based approach. Compared to the single model (i.e., the Skin Doctor CP:Bio model), the advantages of the combined approach with respect to performance are outweighed by the fact that the single model has much lower complexity and, hence, better interpretability.

### 3.7. Investigation of the Influence of Experimental Skin Sensitization Assay Results on Predictivity

Feature selection with Lasso and the RF algorithm identified the three bioactivity descriptors derived from the three skin sensitization-specific assays (i.e., DPRA, KeratinoSens assay, h-CLAT) as important for modeling the LLNA. In order to obtain a better understanding of the role and significance of these three bioactivity descriptors, we investigated them from different perspectives.

First, we determined the (5-fold) CV performance of the CP models for the DPRA, KeratinoSens assay, and h-CLAT descriptors on the (i) 194 compounds measured in the DPRA, (ii) 190 compounds measured in the KeratinoSens assay, and (iii) 160 compounds measured in the h-CLAT. The KeratinoSens and h-CLAT models (Table 6) were valid at a significance level of 0.2 (validities of 0.82 and 0.87, respectively) while the DPRA model showed a slight underperformance (validity 0.74). The efficiencies of the models were fairly low (0.51 to 0.71) in comparison to most of the other CP models for bioactivity prediction. We assume that the low efficiency is related to the fact that the training sets for these CP models are small (<200 compounds). The other evaluated performance measures are within expectations (e.g., MCC between 0.30 and 0.54). Overall, we conclude from these results that the predicted assay outcomes from these three models could make a substantial contribution to models predicting the skin sensitization potential.

Second, we investigated (by 10-fold CV on the full LLNA data set) whether the high importance attributed by Lasso to the skin sensitization-specific assays could be a result of overlaps in the training or test data of the LLNA model (SkinDoctor CP:Bio model) and the training data of the DPRA/KeratinoSens assay/h-CLAT models. For the overlapping compounds, the *p*-values used as bioactivity descriptors should be accurate (since the experimental value of the in vitro assays is known) and therefore more informative. In order to investigate this, we determined the performance of the SkinDoctor CP:Bio model in dependence of the number of compounds overlapping between the LLNA data set (i.e., the test data within each fold) and the training data of the DPRA/KeratinoSens assay/h-CLAT models. We found that six compounds of the LLNA data set were present also in exactly one of the DPRA/KeratinoSens assay/h-CLAT training sets, 45 compounds were present in exactly two of these assays, and 132 compounds in each of these three assays. Note that the number of compounds present in the LLNA data set and in exactly one of the three non-animal assay data sets is too low to make any meaningful observations, for which reason this case was not further pursued. For the remaining two subsets of compounds, the performances of the models were comparable to each other as well as to the subset containing the compounds that are not present in any of three assay data sets (Table 7). For this reason, we are confident that the importance attributed to the predicted DPRA, KeratinoSens assay, and h-CLAT outcomes is genuine and not a result of a bias in the data.

Third, we tested the capacity of a model trained only on DPRA, KeratinoSens assay, and h-CLAT assay data to predict the outcomes of the LLNA. This experiment is particularly interesting because a number of existing in silico models for the prediction of the skin sensitization potential are trained exclusively on data from these three assays [66,67,68].

In five-fold CV, our CP model trained exclusively on DPRA, KeratinoSens assay, and h-CLAT assay data descriptors (n_estimators = 500; all other parameters default) was valid at all error significance levels investigated (Table 8), but its efficiency (0.21 at ε = 0.05; 0.88 at ε = 0.30) and MCC (0.48 at ε = 0.05; 0.37 at ε = 0.30) were substantially lower than those of the CP model derived from the ten selected bioactivity descriptors. These results indicate that the bioactivity descriptors derived from other assays add relevant, additional information to the models that is needed to obtain good classifiers.

### 3.8. Impact of the Limitation of the Available Experimental Data on Model Performance

Most of the freely available models for the prediction of the skin sensitization potential of small molecules are trained on LLNA data, and the evaluation reports for many of these models indicate that their performance is comparable [29,40,44,57]. It is plausible that the observed plateauing of model performance is related to the limited quantity and quality of the data available for model development. In order to investigate whether our classifiers could benefit from additional LLNA data, we investigated the relationship between model performance and the size of the training data.

**Figure 7:**
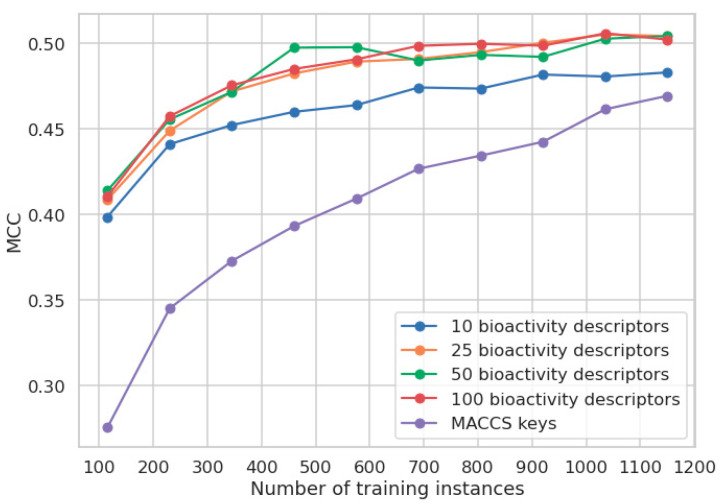
Performance of the RF classifier (n_estimators = 500; all other parameters default) underlying the CP model as a function of the number of instances the model was trained on.

As expected, and shown in Figure 7, the performance of models increases with the number of training instances, regardless of the type of descriptors used. The MCCs of the models based on bioactivity descriptors improve from an average of 0.41 to an average of 0.50, respectively. Consistent with our initial CV experiments, the use of more than ten bioactivity descriptors yields minor improvements in model performance that we believe are outweighed by higher model complexity.

The MCC of the model based on MACCS keys improves from 0.28 (when trained on 115 compounds) to 0.47 (when trained on 1150 compounds), indicating that the models trained on MACCS keys require substantially more training data than the models trained on bioactivity descriptors to obtain good performance. In this particular case, the MACCS keys model reaches a comparable performance to the model based on bioactivity descriptors only when all the available LLNA data are used for modeling. This leaves the MACCS keys model clearly more data-hungry than the models based on predicted bioactivities, with the benefit of showing the potential to surpass the model based on predicted bioactivities given the availability of sufficient amounts of data.

## 4. Conclusions

In this work, we report on the development and validation of a new machine learning model for the prediction of the skin sensitization potential of small organic molecules: Skin Doctor CP:Bio. Whereas the previously reported models are mostly based on molecular fingerprints (which in general are difficult to interpret), Skin Doctor CP:Bio utilizes just ten bioactivity descriptors to reach competitive performance. Most of these bioactivity descriptors are known to be directly or indirectly linked to skin sensitization, which adds to the interpretability of the model and supports its meaningfulness.

At the significance level of 0.20, Skin Doctor CP:Bio obtained an efficiency of 0.82 and an MCC of 0.53 on the holdout data set of 257 compounds. These results demonstrate the good performance of the model and, hence, the relevance of the selected bioactivity descriptors. Analysis of the LLNA training data projected into the new feature space proves that cosmetics, drugs, and agrochemicals are well embedded in the data, hence corroborating the relevance of the model to different industries.

In an attempt to further improve model performance and coverage, we explored different strategies to exploit the information contained in molecular fingerprints (MACCS keys) and biological descriptors. The models obtained from these experiments showed minor improvements in performance that are outweighed by the costs of higher model complexity and limited interpretability.

An important observation to make was that models based on MACCS keys are clearly more data-hungry than models based on predicted bioactivities. Only when using all of the available LLNA data, the model based on MACCs keys was able to catch up with the model based on predicted bioactivities. This highlights the relevance of the presented approach to the development of strategies to address the many questions in biology, pharmacology, and toxicology where measured data are scarce. We believe that the modeling strategies presented in this work could be easily adopted to address many of these research questions. The Skin Doctor CP:Bio model is available free of charge for academic research purposes.

## Figures and Tables

**Table 1:** Ten-fold CV Performance of Models Based on 10 Bioactivity Descriptors ^1^.

Error Significance ε	Validity	Efficiency	ACC	MCC	CCR	Sens	Spec	NPV	PPV
0.05	0.95 (0.03)	0.38 (0.06)	0.88 (0.06)	0.76 (0.12)	0.88 (0.05)	0.87 (0.08)	0.89 (0.08)	0.92 (0.06)	0.85 (0.11)
0.10	0.89 (0.03)	0.61 (0.06)	0.83 (0.05)	0.66 (0.10)	0.83 (0.05)	0.83 (0.10)	0.83 (0.08)	0.88 (0.07)	0.77 (0.09)
0.20	0.79 (0.05)	0.86 (0.04)	0.76 (0.06)	0.51 (0.12)	0.76 (0.06)	0.75 (0.09)	0.77 (0.08)	0.82 (0.07)	0.69 (0.07)
0.30	0.69 (0.07)	0.92 (0.03)	0.74 (0.06)	0.47 (0.11)	0.74 (0.06)	0.72 (0.08)	0.75 (0.07)	0.79 (0.06)	0.67 (0.06)

^1^ Standard deviation in parentheses.

**Table 2:** Overview of the Top-10 Bioactivity Descriptors.

Descriptor Name	Assay Title	Mean Lasso Coefficient ¹	σ (Lasso Coefficient)	Correlation to Positive LLNA Outcome ²	5-Fold CV Performance at Significance Level of 0.20			Most Correlating Assays ³
					Validity	Efficiency	MCC	
p0 BSK KF3CT ICAM1 down	Bioseek human keratinocytes and foreskin fibroblasts intercellular adhesion molecule 1 assay	0.074	0.009	positive	0.80	0.83	0.41	BSK KF3CT SRB down (0.79)
								BSK KF3CT TGFb1 down (0.78)
								BSK KF3CT MCP1 down (0.78)
								BSK KF3CT uPA down (0.78)
								BSK hDFCGF TIMP1 down (0.77)
p1 BSK 4H uPAR down	Bioseek human umbilical vein endothelium plasminogen activator, urokinase receptor assay	0.051	0.045	negative	0.81	0.82	0.46	BSK 3C uPAR down (0.83)
								BSK LPS SRB down (0.81)
								BSK 3C MCP1 down (0.81)
								BSK 4H SRB down (0.8)
								BSK SAg MCP1 down (0.8)
p0 Chromosome aberration	Chromosome aberration assay	0.049	0.010	positive	0.79	0.70	0.30	Mammalian cell gene mutation (0.47)
								AMES (0.41)
								Inhibitors of Hepatocyte nuclear factor 4 (HNF4) dimerization (0.35)
								Modulator of Muscarinic acetylcholine receptor M4 (−0.33)
								Modulator of Bradykinin B2 receptor (−0.33)
p1 DPRA	Direct peptide reactivity assay	0.047	0.013	positive	0.74	0.71	0.30	h-CLAT (0.42)
								Inhibitors of Hepatocyte nuclear factor 4 (HNF4) dimerization (0.31)
								KeratinoSens (0.31)
								Inhibit CYP2C19 Activity (−0.29)
								Modulator of Peroxisome proliferator-activated receptor gamma (−0.29)
p1 Modulator of Dopamine D1 receptor	Modulator of Dopamine D1 receptor assay	0.045	0.006	positive	0.81	0.81	0.98	Modulator of Alpha-2b adrenergic receptor (0.37)
								Modulator of Serotonin 1a (5-HT1a) receptor (0.32)
								Modulator of Alpha-2a adrenergic receptor (0.31)
								Modulator of Serotonin 2a (5-HT2a) receptor (0.31)
								Modulators of myocardial damage (0.3)
p1 h-CLAT	Human cell line activation test	0.043	0.013	positive	0.87	0.56	0.54	PGPinhibition (−0.48)
								Caco2 (0.46)
								LTEA HepaRG DDIT3 up (−0.46)
								ATG TA CIS up (−0.46)
								Modulator of P2X purinoceptor 3 (−0.45)
p1 BSK 3C E-selectin down	Bioseek human umbilical vein endothelium selectin E assay	0.043	0.021	positive	0.79	0.77	0.41	BSK 3C VCAM1 down (0.82)
								BSK 4H Pselectin down (0.81)
								BSK 4H VCAM1 down (0.81)
								BSK 3C MCP1 down (0.81)
								BSK 4H SRB down (0.79)
p1 LTEA HepaRG APOA5 dn	LifeTech/Expression Analysis human HepaRG apolipoprotein A-V assay	0.040	0.012	negative	0.82	0.77	0.51	LTEA HepaRG CYP4A22 dn (0.78)
								LTEA HepaRG CYP4A11 dn (0.77)
								LTEA HepaRG FMO3 dn (0.76)
								LTEA HepaRG HMGCS2 dn (0.76)
								LTEA HepaRG GSTA2 dn (0.75)
p1 KeratinoSens	ARE-Nrf2 Luciferase test method	0.039	0.004	positive	0.82	0.51	0.31	DPRA (0.31)
								h-CLAT (0.31)
								Inhibitors of Hepatocyte nuclear factor 4 (HNF4) dimerization (0.29)
								Inhibit CYP1A2 Activity (0.27)
								Modulator of Monoamine oxidase A (0.27)
p0 ATG NRF2 ARE CIS up	Attagene human HepG2 nuclear factor, erythroid 2-like 2 assay	0.036	0.014	positive	0.81	0.87	0.55	ATG PPARg TRANS up (0.67)
								ATG VDRE CIS up (0.66)
								ATG MRE CIS up (0.65)
								ATG PXR TRANS up (0.64)
								ATG AP 1 CIS up (0.64)

^1.^Mean over the 10 folds of the CV. Note that the feature importance rankings of the Lasso model and the RF model may differ. ^2.^Correlation of the positive assay outcomes and the skin sensitization potentials measured in the LLNA. Since the probability of a compound to belong to the inactive class (p0) or the active class (p1) in a given assay are strongly correlated, either p0 or p1 is selected as an important descriptor by the Lasso model for that assay. Depending on whether p0 or p1 has been selected, and depending on the algebraic sign of the mean Lasso coefficient, a positive predicted assay outcome can either be associated with a positive or a negative LLNA result (i.e., if p0 has a positive correlation with the LLNA result this describes anticorrelation between the positive outcome of both endpoints). ^3.^Numbers in parentheses report the Kendall τ correlation coefficients between the descriptor and the (most) correlated assay. The full names of the assays are provided in Table S2.

**Table 3:** Performance of the model based on ten selected bioactivity descriptors on the test set.

Error Significance ε	Validity	Efficiency	ACC	MCC	CCR	Sens	Spec	NPV	PPV
0.05	0.95	0.39	0.86	0.72	0.86	0.84	0.88	0.88	0.84
0.10	0.89	0.56	0.81	0.62	0.81	0.85	0.77	0.88	0.74
0.20	0.81	0.82	0.76	0.53	0.77	0.80	0.74	0.83	0.69
0.30	0.70	0.95	0.74	0.49	0.75	0.78	0.72	0.82	0.67

**Table 4:** Class-wise performance of the model based on ten selected bioactivity descriptors on the test set.

Error Significance ε	Class	Validity	Efficiency
0.05	Non-sensitizer	0.95	0.38
	Sensitizer	0.93	0.41
0.10	Non-sensitizer	0.87	0.55
	Sensitizer	0.92	0.58
0.20	Non-sensitizer	0.79	0.82
	Sensitizer	0.83	0.82
0.30	Non-sensitizer	0.67	0.94
	Sensitizer	0.75	0.95

**Table 5:** Performance of the consensus and the combined models on the test set.

**Consensus Model Based on a Set of Rules**									
**Error significance ε ¹**		**Coverage**	**ACC**	**MCC**	**CCR**	**Sens**	**Spec**	**NPV**	**PPV**
0.05		0.51	0.86	0.72	0.86	0.84	0.88	0.88	0.84
0.10		0.71	0.79	0.59	0.80	0.83	0.77	0.88	0.70
0.20		0.89	0.77	0.54	0.78	0.82	0.73	0.85	0.68
0.30		0.83	0.78	0.56	0.79	0.85	0.72	0.88	0.68
**Combined Model Based on Mean *p*-Values**									
**Error significance ε**	**Validity**	**Efficiency**	**ACC**	**MCC**	**CCR**	**Sens**	**Spec**	**NPV**	**PPV**
0.05	0.97	0.24	0.89	0.77	0.89	0.92	0.86	0.94	0.82
0.10	0.93	0.46	0.86	0.72	0.87	0.94	0.80	0.95	0.76
0.20	0.82	0.79	0.77	0.56	0.78	0.85	0.72	0.87	0.69
0.30	0.71	0.95	0.75	0.50	0.76	0.80	0.72	0.84	0.66

^1^ Error significance of the underlying model, not of the combined model itself.

**Table 6:** Five-fold CV performance of the CP models for DPRA, KeratinoSens assay, and h-CLAT at the significance level of 0.20 ^1^.

Assay to be Predicted	No. Compounds in Data Set	Validity	Efficiency	ACC	ACC (Sensitizers)	ACC (Non-Sensitizers)	F1 Score	MCC
DPRA	194	0.74 (0.09)	0.71 (0.14)	0.64 (0.07)	0.60 (0.06)	0.69 (0.20)	0.64 (0.07)	0.30 (0.18)
KeratinoSens	190	0.82 (0.11)	0.51 (0.08)	0.67 (0.19)	0.66 (0.24)	0.68 (0.23)	0.64 (0.19)	0.31 (0.35)
h-CLAT	160	0.87 (0.03)	0.56 (0.56)	0.78 (0.05)	0.76 (0.15)	0.75 (0.29)	0.74 (0.06)	0.54 (0.08)

^1^ Standard deviation in parentheses.

**Table 7:** Performance of the SkinDoctor CP:Bio model during 10-fold CV on the full LLNA data set in dependence of the number of skin sensitization assays for which experimental data are available.

Error Significance ε	Validity	Efficiency	MCC	Validity	Efficiency	MCC	Validity	Efficiency	MCC
	For Compounds Exclusive to the LLNA Data Set			For Compounds Present in the LLNA Data Set Plus Exactly					
				Two			Three		
				of the DPRA/KeratinoSens Assay/h-CLAT Training Sets					
0.05	0.97	0.33	0.77	0.98	0.44	0.79	0.98	0.45	0.77
0.10	0.91	0.56	0.64	0.96	0.62	0.74	0.93	0.65	0.67
0.20	0.81	0.83	0.52	0.91	0.84	0.66	0.86	0.81	0.51
0.30	0.69	0.94	0.45	0.82	0.93	0.68	0.73	0.93	0.42

**Table 8:** Test set performance of the classifier trained exclusively on predicted values of the DPRA, KeratinoSens and h-CLAT assays.

Error Significance ε	Validity	Efficiency	ACC	MCC	CCR	Sens	Spec	NPV	PPV
0.05	0.94	0.21	0.71	0.48	0.72	0.92	0.52	0.88	0.63
0.10	0.90	0.40	0.75	0.51	0.76	0.82	0.69	0.84	0.67
0.20	0.80	0.70	0.72	0.44	0.72	0.76	0.69	0.81	0.61
0.30	0.72	0.88	0.68	0.37	0.69	0.71	0.66	0.78	0.59

## Data Availability

The models for generating the ten bioactivity descriptors as well as the final LLNA model are available free of charge for academic research from https://doi.org/10.5281/zenodo.5101594, accessed on 7 August 2021 [69]. The LLNA data set used for model development and evaluation was published earlier [29].

## Supplementary Materials

The following are available online at https://www.mdpi.com/article/10.3390/ph14080790/s1. Figure S1. Loadings plot for the PCA on the LLNA and the three reference data sets, based on the ten selected bioactivity descriptors; Table S1: Mean absolute lasso coefficients and standard deviation σ retrieved from the 10-fold CV; Table S2: Full name of the assays with high correlation to the ten selected bioactivity descriptors; Table S3: Comparison of the Skin Doctor CP and Skin Doctor CP:Bio approaches; Table S4: Results of Skin Doctor CP on the test set.

## Abbreviations

ACC	accuracy
CCR	correct classification rate
CP	conformal prediction
CV	cross validation
DPRA	direct peptide reactivity assay
GPMT	guinea pig maximization test
h-CLAT	human cell line activation test
IATA	integrated approach for testing and assessment
LLNA	local lymph node assay
MCC	Matthews correlation coefficient
NPV	negative predictive rate
PC	principal component
PCA	principal component analysis
PPV	positive predictive rate
(Q)SAR	(quantitative) structure activity relationship
RF	random forest
Sens	sensitivity
Spec	specificity
