# Supplementary material for: Predicting the Skin Sensitization Potential of Small Molecules with Machine Learning Models Trained on Biologically Meaningful Descriptors
Source: Pharmaceuticals (Basel). 2021 Aug 11;14(8):790. doi: 10.3390/ph14080790 (PMC8402010; doi:10.3390/ph14080790)
Supplement: Supplementary file 1 [file pharmaceuticals-14-00790-s001.zip › pharmaceuticals-1320189-supplementary.pdf]

Supporting Information

Predicting the Skin Sensitization Potential of Small Molecules with Machine Learning Models Trained on Biologically Meaningful Descriptors

Anke Wilm [1,2], Marina Garcia de Lomana [3], Conrad Stork [1], Neann Mathai [4], Steffen Hirte [3], Ulf Norinder [5,6,7], Jochen Kühnl [8] and Johannes Kirchmair [1,3*]

[1] Center for Bioinformatics (ZBH), Department of Informatics, Universität Hamburg, 20146 Hamburg, Germany; wilm@zbh.uni-hamburg.de (A.W.); stork@zbh.uni-hamburg.de (C.S.)
[2] HITeC e.V., 22527 Hamburg, Germany
[3] Department of Pharmaceutical Sciences, Faculty of Life Sciences, University of Vienna, 1090 Vienna, Austria; a11853333@unet.univie.ac.at (M.G.d.L.); steffen.hirte@univie.ac.at (S.H.)
[4] Computational Biology Unit (CBU), Department of Chemistry, University of Bergen, N-5020 Bergen, Norway; neann.mathai@uib.no
[5] MTM Research Centre, School of Science and Technology, Örebro University, SE-70182 Örebro, Sweden; ulf.norinder@farmbio.uu.se
[6] Department of Computer and Systems Sciences, Stockholm University, SE-16407 Kista, Sweden
[7] Department of Pharmaceutical Biosciences, Uppsala University, SE-75124 Uppsala, Sweden
[8] Front End Innovation, Beiersdorf AG, 22529 Hamburg, Germany; Jochen.Kuehnl@Beiersdorf.com
* Correspondence: johannes.kirchmair@univie.ac.at; Tel.: +43-1-4277-55104

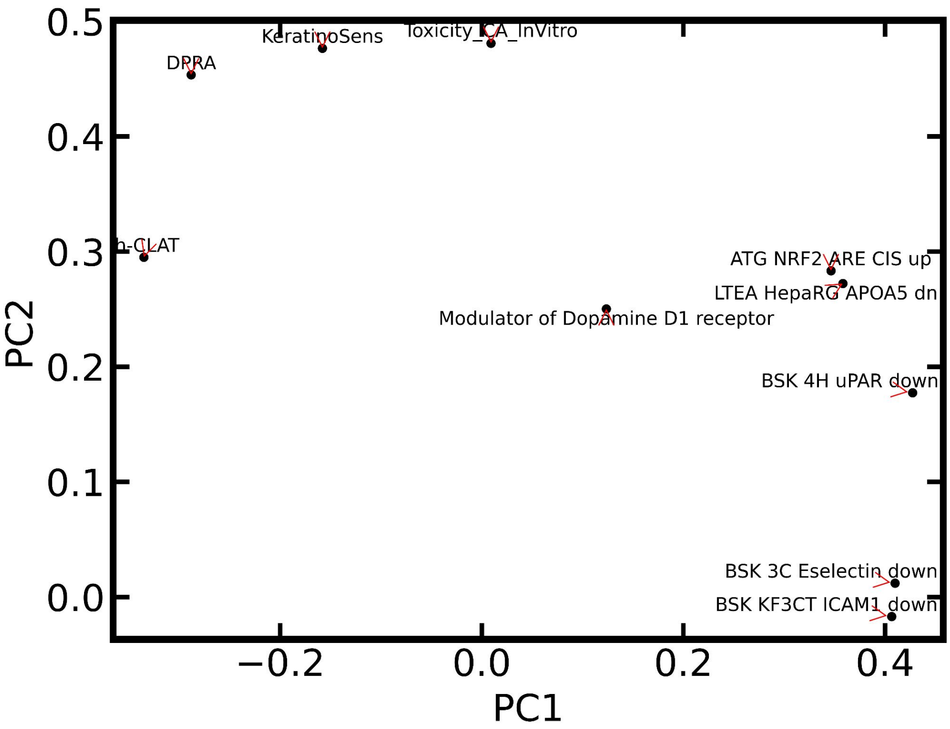

Figure S1. Loadings plot for the PCA on the LLNA and the three reference data sets, based on the ten selected bioactivity descriptors.

Table S1. Mean absolute Lasso coefficients and standard deviation σ retrieved from the 10-fold cross-validation.

Assay name	Mean Lasso coefficient	σ(Lasso coefficient)	Correlation to positive assay outcome
p0 BSK KF3CT ICAM1 down	0.074	0.0088	positive
p1 BSK 4H uPAR down	0.051	0.0454	negative
p0 CA	0.049	0.0096	positive
p1 DPRA	0.047	0.0125	positive
p1 Modulator of Dopamine D1 receptor	0.045	0.0064	positive
p1-h-CLAT	0.043	0.0134	positive
p1 BSK 3C Eselectin down	0.043	0.0210	positive
p1 LTEA HepaRG APOA5 dn	0.040	0.0123	negative
p1-KeratinoSens	0.039	0.0036	positive
p0 ATG NRF2 ARE CIS up	0.036	0.0142	positive
p0 Modulator of Muscarinic acetylcholine receptor M1	0.036	0.0145	positive
p0 Inhibitors and Substrates of Cytochrome P450 2C9	0.032	0.0064	positive

p1 OT ER ERaERb 1440	0.026	0.0129	positive
p1 AMES	0.026	0.0098	positive
p1 LTEA HepaRG FABP1 dn	0.025	0.0144	negative
p1 BSK hDFCGF IP10 down	0.025	0.0200	positive
p1 Activators of the human pregnane X receptor (PXR) signaling pathway	0.025	0.0164	negative
p0 TOX21 RAR LUC Agonist	0.022	0.0095	negative
p1 BSK LPS TNFa down	0.022	0.0212	negative
p1 TOX21 MMP ratio up	0.022	0.0125	negative
p1 TOX21 ERa BLA Agonist ratio	0.021	0.0154	negative
p1 UPITT HCI U2OS AR TIF2 Nucleoli Antagonist	0.021	0.0145	positive
p0 Modulator of Muscarinic acetylcholine receptor M4	0.020	0.0074	positive
p0 OT AR ARSRC1 0480	0.020	0.0204	positive
p1 Modulator of Melatonin receptor 1B	0.019	0.0069	negative
p1 LTEA HepaRG ABCB1 up	0.019	0.0100	negative
p0 Induce genoin human embryonic kidney cells	0.019	0.0092	negative
p1 TOX21 HDAC Inhibition	0.018	0.0156	positive
p0 Modulator of Monoamine oxidase A	0.018	0.0044	positive
p0 TOX21 TR LUC GH3 Antagonist	0.017	0.0234	positive
p0 Mutagenicity	0.016	0.0134	negative
p0 LTEA HepaRG CYP2E1 dn	0.015	0.0167	positive
p1 ATG RORE CIS up	0.015	0.0107	negative
p1 ATG DR4 LXR CIS dn	0.014	0.0092	positive
p1 Modulator of Androgen Receptor	0.013	0.0070	negative
p1 Differential cyto(isogenic chicken DT40 Rev3 mutant cell line)	0.013	0.0104	positive
p1 Block Bile Salt Export Pump	0.013	0.0103	negative
p0 Modulator of Adenosine A1 receptor	0.013	0.0066	negative
p0 Agonist of the AP-1 signaling pathway	0.013	0.0166	positive
p1 LTEA HepaRG CYP1A1 up	0.012	0.0093	positive
p1 Inhibitors and Substrates of Cytochrome P450 2D6	0.012	0.0111	positive
p1 TOX21 FXR BLA antagonist ratio	0.011	0.0182	positive
p0 UPITT HCI U2OS AR TIF2 Nucleoli Agonist	0.011	0.0142	negative

p0 LTEA HepaRG CYP1A2 up	0.011	0.0087	positive
p0 BSK 3C Eselectin down	0.011	0.0141	positive
p0 Modulator of Platelet activating factor receptor	0.011	0.0072	negative
p0 NHEERL ZF 144hpf TERATOSCORE up	0.011	0.0077	positive
p1 Agonist of the RXR signaling pathway	0.010	0.0074	negative
p1 TOX21 AP1 BLA Agonist ratio	0.010	0.0138	negative
p0 TOX21 PR BLA Antagonist ratio	0.010	0.0125	negative
p1 Caco2	0.009	0.0113	positive
p1 BSK hDFCGF MCSF down	0.009	0.0069	positive
p1 Differential cytoagainst isogenic chicken DT40 cell lines with known DNA damage response pathways Rad54Ku70 mutant cell line	0.008	0.0086	positive
p1 TOX21 AhR LUC Agonist	0.008	0.0107	negative
p0 NCCT HEK293T CellTiterGLO	0.008	0.0096	positive
p0 Antagonist of the retinoic acid receptor (RAR) signaling pathway	0.008	0.0108	negative
p1 TOX21 ERa LUC VM7 Agonist	0.008	0.0036	negative
p1 ATG RXRb TRANS up	0.007	0.0065	positive
p1 TOX21 MMP ratio down	0.007	0.0127	positive
p1 Modulator of Calcitonin gene-related peptide type 1 receptor	0.007	0.0061	positive
p0 Modulator of Glutamate NMDA receptor	0.007	0.0067	negative
p0 Modulator of Neurokinin 2 receptor	0.007	0.0066	negative
p1 BSK hDFCGF TIMP1 down	0.007	0.0139	positive
p0 Modulator of Adenosine A3 receptor	0.007	0.0101	negative
p1 ATG NRF2 ARE CIS up	0.006	0.0079	positive
p1 Modulator of Dopamine transporter	0.006	0.0063	positive
p1 ATG Ets CIS dn	0.006	0.0084	negative
p0 Cytoin HepG2 cells 40 hour	0.006	0.0106	negative
p1 ATG PBREM CIS up	0.006	0.0101	negative
p0 Inhibit CYP1A2 Activity	0.006	0.0119	positive
p1 LTEA HepaRG ALPP dn	0.006	0.0169	negative
p1 CA	0.006	0.0104	positive

p0 Modulator of Neuronal acetylcholine receptor alpha4beta2	0.006	0.0072	positive
p0 Block Bile Salt Export Pump	0.005	0.0108	negative
p1 TOX21 RXR BLA Agonist ratio	0.005	0.0060	negative
p1 BSK BE3C IL1a down	0.005	0.0141	negative
p0 Modulator of Melatonin receptor 1B	0.005	0.0040	negative
p1 ATG HIF1a CIS up	0.005	0.0053	negative
p0 Modulator of Receptor protein-tyrosine kinase erbB-2	0.005	0.0084	positive
p0 OT ER ERaERb 1440	0.005	0.0117	positive
p0 Modulator of Cholecystokinin A receptor	0.005	0.0051	negative
p1 Disruptors of the mitochondrial membrane potential	0.005	0.0069	positive
p0 Modulator of Sodium channel protein type IX alpha subunit	0.004	0.0046	negative
p1 UPITT HCI U2OS AR TIF2 Nucleoli Agonist	0.004	0.0065	negative
p0 BSK CASM3C MCP1 down	0.004	0.0074	positive
p0 Modulator of GABA-A receptor alpha-1beta-3gamma-2	0.003	0.0059	negative
p0 LTEA HepaRG CYP1A1 up	0.003	0.0075	positive
p0 Modulator of Neuronal acetylcholine receptor protein alpha-7 subunit	0.003	0.0060	negative
p0 Cytoin HepG2 cells 32 hour	0.003	0.0070	negative
p1 Modulator of Sodium channel protein type IX alpha subunit	0.003	0.0033	negative
p1 ATG C EBP CIS up	0.003	0.0055	negative
p1 Modulator of Acetylcholinesterase	0.003	0.0034	positive
p1 BSK hDFCGF Proliferation down	0.003	0.0044	positive
p1 OT FXR FXRSRC1 1440	0.003	0.0085	negative
p0 Modulator of Serotonin 7 (5-HT7) receptor	0.003	0.0050	positive
p1 Modulator of GABA-A receptor alpha-2beta-3gamma-2	0.003	0.0038	negative
p1 Antagonist of the estrogen receptor alpha (ER-alpha) signaling pathway	0.003	0.0052	negative
p1 ATG E Box CIS dn	0.003	0.0080	positive
p1 Modulator of Serotonin 2b (5-HT2b) receptor	0.003	0.0046	negative

p1 ATG ERa TRANS up	0.003	0.0039	positive
p1 TOX21 TSHR Agonist ratio	0.002	0.0061	positive
p1 Modulator of Serotonin 7 (5-HT7) receptor	0.002	0.0026	negative
p0 Modulator of Dopamine transporter	0.002	0.0044	positive
p1 BSK SAg CD69 down	0.002	0.0068	positive
p1 ATG BRE CIS up	0.002	0.0040	negative
p1 ACEA ER 80hr	0.002	0.0052	negative
p1 Modulator of Adenosine A1 receptor	0.002	0.0032	negative
p1 APR HepG2 CellLoss 72h dn	0.002	0.0059	negative
p0 Activators of the human pregnane X receptor (PXR) signaling pathway	0.002	0.0043	negative
p0 Modulator of Norepinephrine transporter	0.002	0.0030	positive
p0 Modulator of Vascular endothelial growth factor receptor 2	0.002	0.0054	positive
p0 BSK CASM3C MCSF down	0.002	0.0029	positive
p1 Modulator of Alpha-1a adrenergic receptor	0.002	0.0035	positive
p1 BSK hDFCGF CollagenIII down	0.002	0.0034	positive
p0 Modulator of Serotonin 2b (5-HT2b) receptor	0.002	0.0030	negative
p0 Modulators of myocardial damage	0.002	0.0026	positive
p0 Modulator of HERG	0.002	0.0048	negative
p1 BSK CASM3C MCSF down	0.002	0.0048	positive
p1 ATG PXR TRANS up	0.002	0.0048	positive
p1 Modulator of Alpha-2a adrenergic receptor	0.002	0.0024	positive
p0 Modulator of Serotonin 1b (5-HT1b) receptor	0.002	0.0037	negative
p0 Modulator of Peroxisome proliferator-activated receptor gamma	0.001	0.0041	negative
p1 Modulator of P2X purinoceptor 7	0.001	0.0019	negative
p0 Modulator of Cannabinoid CB2 receptor	0.001	0.0043	positive
p0 Modulator of P2X purinoceptor 3	0.001	0.0042	positive
p1 Activator the aryl hydrocarbon receptor (AhR) signaling pathway	0.001	0.0028	negative
p1 Modulator of Serotonin 1b (5-HT1b) receptor	0.001	0.0027	negative
p1 ATG PPARg TRANS up	0.001	0.0028	positive
p0 Modulator of Delta opioid receptor	0.001	0.0032	positive

p1 ATG ISRE CIS dn	0.001	0.0025	negative
p1 Modulator of Histamine H1 receptor	0.001	0.0024	positive
p1 Modulator of Platelet-derived growth factor receptor beta	0.001	0.0026	positive
p1 ACEA AR antagonist 80hr	0.001	0.0035	negative
p1 DIO1	0.001	0.0032	positive
p0 Differential cytoagainst isogenic chicken DT40 cell lines with known DNA damage response pathways Rad54Ku70 mutant cell line	0.001	0.0033	positive
p0 Modulator of Calcitonin gene-related peptide type 1 receptor	0.001	0.0032	negative
p1 TOX21 ERR Agonist	0.001	0.0032	positive
p1 TOX21 DT40	0.001	0.0032	positive
p1 Modulator of Neuronal acetylcholine receptor alpha4beta2	0.001	0.0014	negative
p0 Caco2	0.001	0.0032	positive
p1 TOX21 AR LUC MDAKB2 Agonist	0.001	0.0032	negative
p1 Inhibitors of Hepatocyte nuclear factor 4 (HNF4) dimerization	0.001	0.0031	positive
p0 Modulator of Neurokinin 1 receptor	0.001	0.0029	negative
p1 Modulator of Adenosine A2a receptor	0.001	0.0026	negative
p1 Antagonist of the farnesoid-X-receptor (FXR) signaling pathway	0.001	0.0021	negative
p1 Modulator of Dopamine D2 receptor	0.001	0.0020	positive
p0 AMES	0.001	0.0014	positive
p0 LTEA HepaRG UGT1A1 up	0.001	0.0018	positive
p1 Modulator of GABA-A receptor alpha-1beta-3gamma-2	0.001	0.0011	negative
p0 TOX21 PGC ERR Agonist	0.001	0.0016	negative
p1 TOX21 CAR Agonist	0.001	0.0016	negative
p1 TOX21 DT40 657	0.001	0.0012	positive
p0 Modulator of Angiotensin-converting enzyme	0.001	0.0016	positive
p1 Antagonist of the vitamin D receptor (VDR) signaling pathway	0.001	0.0015	positive
p1 Modulator of Serotonin 4 (5-HT4) receptor	0.001	0.0011	negative

p0 ATG DR4 LXR CIS dn	0.000	0.0015	positive
p0 TOX21 TSHR Agonist ratio	0.000	0.0014	positive
p0 TOX21 MMP ratio up	0.000	0.0014	negative
p1 Modulator of GABA-A receptor alpha-5beta-3gamma-2	0.000	0.0014	negative
p1 ATG TA CIS up	0.000	0.0012	negative
p1 Modulator of Alpha-1b adrenergic receptor	0.000	0.0012	positive
p1 Agonist of H2AX	0.000	0.0012	positive
p1 Modulator of Urotensin II receptor	0.000	0.0012	negative
p1 Modulator of Adenosine A3 receptor	0.000	0.0012	negative
p0 MammMutagenicity	0.000	0.0011	positive
p0 Modulator of Serotonin 4 (5-HT4) receptor	0.000	0.0011	positive
p0 LTEA HepaRG CYP7A1 dn	0.000	0.0010	positive
p0 TOX21 HSE BLA agonist ratio	0.000	0.0009	negative
p0 BSK CASM3C VCAM1 down	0.000	0.0009	positive
p0 Bioavailability	0.000	0.0009	negative
p1 Modulator of Serotonin transporter	0.000	0.0008	positive
p1 Induce genoin human embryonic kidney cells	0.000	0.0008	negative
p0 Modulator of Alpha-1a adrenergic receptor	0.000	0.0006	negative
p1 Antagonist of the androgen receptor (AR) signaling pathway dup	0.000	0.0006	negative
p0 BSK hDFCGF IP10 down	0.000	0.0006	positive
p1 Modulator of Angiotensin-converting enzyme	0.000	0.0006	positive
p0 Modulator of Sigma opioid receptor	0.000	0.0006	positive
p1 BSK 4H MCP1 down	0.000	0.0005	positive
p0 Modulator of Vascular endothelial growth factor receptor 3	0.000	0.0004	negative
p0 BSK KF3CT TGFb1 down	0.000	0.0004	positive
p1 ATG NF kB CIS dn	0.000	0.0003	positive
p0 Modulator of Serotonin 3a (5-HT3a) receptor	0.000	0.0003	negative
p1 ATG RARa TRANS dn	0.000	0.0003	positive
p1 TOX21 p53 BLA p2 ratio	0.000	0.0002	positive
p1 Modulator of Cannabinoid CB2 receptor	0.000	0.0002	positive
p1 Cytoin HEK293 cells 32 hour	0.000	0.0002	positive

p1 Modulator of Serotonin 1a (5-HT1a) receptor	0.000	0.0001	negative
p1 Modulator of Sigma opioid receptor	0.000	0.0001	positive
p0 Modulator of P2X purinoceptor 7	0.000	0.0001	negative
p0 Modulator of TNF-alpha	0.000	0.0001	negative
p1 Antagonist of the estrogen receptor alpha (ER-alpha) signaling pathway dup	0.000	0.0001	negative
p0 ATG ISRE CIS dn	0.000	0.0000	negative
p1 Inhibitors and Substrates of Cytochrome P450 3A4	0.000	0.0000	negative

Table S2. Full name of the assays with high correlation to the ten selected bioactivity descriptors.

Descriptor Name	Assay title
AMES	Ames test for mammalian environmental mutagenicity
Caco2	Caco-2 permeability assay to investigate intestinal permeability
Inhibit CYP1A2 Activity	Inhibitors of CYP1A2 activity assay
Inhibit CYP2C19 Activity	Inhibitors of CYP2C19 activity assay
Inhibitors of Hepatocyte nuclear factor 4 (HNF4) dimerization	Inhibitors of Hepatocyte nuclear factor 4 (HNF4) dimerization assay
Modulator of Alpha-2a adrenergic receptor	Modulator of alpha-2a adrenergic receptor assay
Modulator of Alpha-2b adrenergic receptor	Modulator of alpha-2b adrenergic receptor assay
Modulator of Bradykinin B2 receptor	Modulator of bradykinin B2 receptor assay
Modulator of Monoamine oxidase A	Modulator of monoamine oxidase A assay
Modulator of Muscarinic acetylcholine receptor M4	Modulator of muscarinic acetylcholine receptor M4 assay
Modulator of P2X purinoceptor 3	Modulator of P2X purinoceptor 3 assay
Modulator of Peroxisome proliferator-activated receptor gamma	Modulator of peroxisome proliferator-activated receptor gamma assay
Modulator of Serotonin 1a (5-HT1a) receptor	Modulator of serotonin 1a (5-HT1a) receptor assay
Modulator of Serotonin 2a (5-HT2a) receptor	Modulator of serotonin 2a (5-HT2a) receptor assay
Modulators of myocardial damage	Modulators of myocardial damage assay
MammMutagenicity	Mammalian cell gene mutation assay
PGPinhibition	P-glycoprotein (Pgp) inhibition assay
ATG AP 1 CIS up	Attagene human HepG2 FBJ murine osteosarcoma viral oncogene homolog | jun proto-oncogene assay
ATG MRE CIS up	Attagene human HepG2 metal-regulatory transcription factor 1 assay
ATG PPARg TRANS up	Attagene TRANS-FACTORIAL HepG2 Human Peroxisome Proliferator-activated Receptor Gamma (PPARg) Activation Assay
ATG PXR TRANS up	Attagene human HepG2 nuclear receptor subfamily 1, group I,

	member 2 assay
ATG TA CIS up	Attagene human HepG2 unspecified assay
ATG VDRE CIS up	Attagene human HepG2 vitamin D (1,25-dihydroxyvitamin D3) receptor assay
BSK 3C MCP1 down	Bioseek human umbilical vein endothelium chemokine (C-C motif) ligand 2 assay
BSK 3C uPAR down	Bioseek human umbilical vein endothelium plasminogen activator, urokinase receptor assay
BSK 3C VCAM1 down	Bioseek human umbilical vein endothelium vascular cell adhesion molecule 1 assay
BSK 4H Pselectin down	Bioseek human umbilical vein endothelium selectin P (granule membrane protein 140kDa, antigen CD62) assay
BSK 4H SRB down	Bioseek human umbilical vein endothelium selectin P (granule membrane protein 140kDa, antigen CD62) assay
BSK 4H VCAM1 down	Bioseek human umbilical vein endothelium vascular cell adhesion molecule 1 assay
BSK hDFCGF TIMP1 down	Bioseek human foreskin fibroblast TIMP metallopeptidase inhibitor 1 assay
BSK KF3CT MCP1 down	Bioseek human keratinocytes and foreskin fibroblasts chemokine (C-C motif) ligand 2 assay
BSK KF3CT SRB down	Bioseek human keratinocytes and foreskin fibroblasts unspecified assay
BSK KF3CT TGFb1 down	Bioseek human keratinocytes and foreskin fibroblasts transforming growth factor, beta 1 assay
BSK KF3CT uPA down	Bioseek human keratinocytes and foreskin fibroblasts plasminogen activator, urokinase assay
BSK LPS SRB down	Bioseek human umbilical vein endothelium and peripheral blood mononuclear cells unspecified assay
BSK SAg MCP1 down	Bioseek human umbilical vein endothelium and peripheral blood mononuclear cells chemokine (C-C motif) ligand 2 assay
LTEA HepaRG CYP4A11 dn	LifeTech/Expression Analysis human HepaRG cytochrome P450, family 4, subfamily A, polypeptide 11 assay
LTEA HepaRG CYP4A22 dn	LifeTech/Expression Analysis human HepaRG cytochrome P450, family 4, subfamily A, polypeptide 22 assay
LTEA HepaRG DDIT3 up	LifeTech/Expression Analysis human HepaRG DNA-damage-inducible transcript 3 assay
LTEA HepaRG FMO3 dn	LifeTech/Expression Analysis human HepaRG flavin

	containing monooxygenase 3 assay
LTEA HepaRG GSTA2 dn	LifeTech/Expression Analysis human HepaRG glutathione S-transferase alpha 2 assay
LTEA HepaRG HMGCS2 dn	LifeTech/Expression Analysis human HepaRG 3-hydroxy-3-methylglutaryl-CoA synthase 2 (mitochondrial) assay

Table S3: Comparison of the Skin Doctor CP and Skin Doctor CP:Bio approaches.

	**Skin Doctor CP**	**Skin Doctor CP:Bio**
type of descriptors	MACCS Keys	Bioactivity descriptors
number of descriptors	166	10
n estimators	1000	500
max features	"sqrt"	"auto"
random state	43	43
number of compounds in the test set	257	257
number of compounds in the training set	1028	1021

Table S4: Results of Skin Doctor CP on the test set.

Significance level ε	Validity	Efficiency	ACC	MCC	CCR	SE	SP	NPV	PPV
0.05	0.96	0.32	0.89	0.78	0.89	0.91	0.88	0.94	0.83
0.10	0.91	0.49	0.83	0.66	0.84	0.90	0.78	0.92	0.72
0.20	0.82	0.79	0.77	0.55	0.78	0.84	0.72	0.88	0.65
0.30	0.69	0.92	0.75	0.51	0.76	0.81	0.70	0.84	0.65
